# Heat dissipation in subterranean rodents: the role of body region and social organisation

**DOI:** 10.1038/s41598-021-81404-3

**Published:** 2021-01-21

**Authors:** František Vejmělka, Jan Okrouhlík, Matěj Lövy, Gabriel Šaffa, Eviatar Nevo, Nigel Charles Bennett, Radim Šumbera

**Affiliations:** 1 Department of Zoology, Faculty of Science, University of South Bohemia, 370 05 České Budějovice, Czech Republic; 2 Department of Zoology and Entomology, Mammal Research Institute, University of Pretoria, Pretoria, 0002 South Africa; 3 Institute of Evolution, University of Haifa, 3498838 Haifa, Israel

**Keywords:** Ecophysiology, Animal behaviour, Animal physiology, Social evolution

## Abstract

The relatively warm and very humid environment of burrows presents a challenge for thermoregulation of its mammalian inhabitants. It was found that African mole-rats dissipate body heat mainly through their venter, and social mole-rats dissipate more body heat compared to solitary species at lower temperatures. In addition, the pattern of the ventral surface temperature was suggested to be homogeneous in social mole-rats compared to a heterogeneous pattern in solitary mole-rats. To investigate this for subterranean rodents generally, we measured the surface temperatures of seven species with different degrees of sociality, phylogeny, and climate using infrared thermography. In all species, heat dissipation occurred mainly through the venter and the feet. Whereas the feet dissipated body heat at higher ambient temperatures and conserved it at lower ambient temperatures, the ventral surface temperature was relatively high in all temperatures indicating that heat dissipation to the environment through this body region is regulated mainly by behavioural means. Solitary species dissipated less heat through their dorsum than social species, and a tendency for this pattern was observed for the venter. The pattern of heterogeneity of surface temperature through the venter was not related to sociality of the various species. Our results demonstrate a general pattern of body heat exchange through the three studied body regions in subterranean rodents. Besides, isolated individuals of social species are less able to defend themselves against low ambient temperatures, which may handicap them if staying alone for a longer period, such as during and after dispersal events.

## Introduction

The heat exchange between an endotherm and its environment is mediated via radiation, conduction, convection, and evaporation (e.g.^1^). In mammals, the transfer of excess heat to the environment is facilitated by so-called thermal windows. These sparsely furred body regions are usually found at the body extremities, e.g. ear pinnae^2,3^, the feet^3–6^, and the tail^7^, but may also occur on the trunk^8^, and on the ventral body side^9–13^. Together with behavioural regulation, such as posture changes, or seeking convenient microhabitats, heat transfer in these body parts is regulated mainly by vasodilatation and vasoconstriction^1^.

Mammalian thermal biology is influenced by multiple factors, but body size plays a crucial role. Compared to their larger counterparts, small mammals have to compensate for the relatively higher heat losses in cold environments due to their larger surface-to-volume ratio^14^. There are various strategies as to how small mammals can minimise heat loss at low ambient temperature (T_a_). For example, an individual curled up into a ball reduces its surface-to-volume ratio^15–17^. Similarly, huddling as a mechanism of social thermoregulation decreases heat loss rate (e.g.^18–20^). At higher T_a_s, rapid heat dissipation is crucial, especially in situations where animals produce a surplus of metabolic heat, for example due to heavy physical activity. Animals move to a place with a more suitable microclimate or increase their evaporative water loss by sweating and/or panting. Thermal windows may also be actively exposed to the environment at high T_a_s^12,21^.

Mammals living in subterranean burrows face a very challenging environment including difficulties in heat dissipation. The burrow environment is relatively warm and very humid (> 80% relative humidity even in very arid areas) with limited ventilation, and such a combination presents a problem for their thermoregulation^22–25^. In addition, excavation of burrows, which is the main way to reach food, increases metabolic rate up to five times that of resting^22,26–28^. Thus, the surplus body heat related to such increase needs to be effectively dissipated to avoid overheating^22,29^. Although subterranean mammals live in a thermally relatively stable environment whose overall T_a_ does not decrease much below the thermoneutral zone (TNZ), most of them have dense fur that well conserve body heat^30–33^. This may complicate heat offloading at higher T_a_s or after digging. Finally, these mammals have reduced or even lack most body extremities such as tails and ear pinnae used for heat dissipation in non-fossorial mammals.

From this point of view it has for a long time been an enigma as to how subterranean mammals avoid overheating, especially during highly heat-producing activities. It was speculated that the excess metabolic heat is dissipated mainly through the venter in the Talas tuco-tuco *Ctenomys talarum*^34^. This heat dissipation pathway was later supported by the analysis of surface temperatures (T_s_), i.e. temperature of the fur surface facing to the environment, in five African mole-rats (Bathyergidae, Rodentia)^31,35^. Both studies demonstrated that the feet which are generally less haired compared to other body regions in all bathyergids also play a relevant role in heat dissipation. It was also shown that two mole-rat species, the giant mole-rat *Fukomys mechowii* and the silvery mole-rat *Heliophobius argenteocinereus*, cool themselves very effectively, while burrowing via contact of their body with the colder surrounding soil, especially when the soil is wet^36^. Although interspecific differences in heat dissipation in mole-rats could be attributed mainly to the climate of the habitats^35^, remarkable differences were found, surprisingly, also between species from the same climatic conditions and habitats. Thus, social *F. mechowii* has a shorter and sparser fur allowing more effective body heat dissipation and thus higher T_s,_ compared to the solitary *H. argenteocinereus* which has longer and denser fur across most of its body^31^. Higher heat dissipation for social species is disadvantageous at lower T_a_s, but is reduced by huddling^37,38^. Both species also differed in the pattern of T_s_ on the venter. Whereas *F. mechowii* had a uniformly high T_s_ distributed across its venter, *H. argenteocinereus* had a smaller area of high T_s_ on the chest, and the rest of the ventral surface was remarkably colder making the venter heterogeneous in terms of T_s_^31^. However, interpretations regarding the heterogeneity of thermal windows related to differences in social organisation are preliminary because only one solitary and one social species have been compared so far (cf.^31^).

Apart from fur, heat dissipation through a particular body region could be influenced by the characteristics of skin and fat tissue as exhibited in aquatic mammals^39^, or small terrestrial mammals^40^. Nevertheless, to be able to move easily through burrows, mole-rats and probably also other subterranean mammals do not store fat as a subcutaneous layer^41,42^. A recent analysis did not show any remarkable differences in parameters of skin and fat tissue between the dorsum and venter in *F. mechowii* which supports an exclusive role of fur in heat dissipation through the integument^43^. Different fur characteristics can thus have relevant behavioural and ecological consequences in subterranean rodents. For example, it has been suggested that well insulating fur allows *H. argenteocinereus* to colonise the highest altitudes of the Nyika Plateau in Malawi, whereas the sympatric social Whyte’s mole-rat *Fukomys whytei* occurs at lower altitudes^44^. We may predict that individuals of social species, when dispersing singly, have higher thermoregulatory costs due to a less insulating fur because they cannot decrease heat losses by huddling, one of the most efficient energy saving mechanisms in small social mammals^20^. Although it is very difficult to obtain data on the duration of staying alone in social subterranean species in nature, several records indicate solitary individuals of social mole-rats stay for weeks, months or even years on their own^45–48^.

In the current study, we recorded T_s_ using infrared thermography (IRT), and core body temperature (T_b_) to analyse heat dissipation ability, the presence of thermal windows, and the role of social organisation in seven species of subterranean rodents over a T_a_ gradient from 10 to 35 °C. The studied species originated from different phylogenetic lineages (families Bathyergidae, Octodontidae, Spalacidae), climatic zones, and continents with representatives from both social and solitary species (for details see Table 1). As T_b_ may differ among the tested species, we introduce T_diff_ parameter defined as the difference between T_b_ and T_s_ of a particular body region (the dorsum, venter, and feet) at each T_a_ to quantify heat dissipation. Higher values of this parameter indicate lower heat dissipation from the respective body region to its surrounding. Since T_diff_ is determined mainly by the value of T_s_, while T_b_ changes much less, we did not test T_s_ in most analyses to avoid redundant results similar to those of T_diff_. We focused mainly on both extreme T_a_s (10 and 35 °C), and a T_a_ within TNZ of all species (30 °C). We hypothesized that:

**Table 1:** Characteristics of seven studied species of subterranean rodents and their habitats. Means ± SD of body masses are given.

Species (n)	Social organisation	Body mass (g)	TNZ (°C)	Burrow temperature (°C)	Altitude (m a.s.l.)	Locality (GPS)	Climatic zone	AP (mm)	AMT (°C)	MT (°C)	TAR (°C)
*B. suillus* (10)	Solitary	694 ± 146	25–31^50^	12.4–31.6^a^	252	Darling, R. of South Africa (33° 22′ S, 18° 25′ E)	Subtropics	435	17.6	6.7	20.8
*G. capensis* (9)	Solitary	148 ± 48	26.3–34^42^	12.0–30.7^a^ 9.8–36.3^b^ 10.2–29.7^55^	252	Darling, R. of South Africa (33° 22′ S, 18° 25′ E)	Subtropics	435	17.6	6.7	20.8
*C. hottentotus* (10)	Social	71 ± 13	27–30^51^	15.9–27.8^55^	252	Darling, R. of South Africa (33° 22′ S, 18° 25′ E)	Subtropics	435	17.6	6.7	20.8
*F. anselli* (9)	Social	80 ± 11	26–30^53^	18–26^56^^e^	1320	Lusaka, Zambia (15° 28′ S, 28° 25′ E)	Tropics	824	19.9	7.7	22.2
*F.* “Nsanje” (10)	Social	139 ± 27	27–34^52^	–	53	Nsanje, Malawi (16° 55′ S, 35° 16′ E)	Tropics	842	25.7	14	21.8
*S. cyanus* (5)	Social	101 ± 19	26–33^5^	8–32.1^57^	Not known	Not known, Chile	Subtropics/temperate	377	13.1	1.9	22.0
*N. galili* (20)	Solitary	171 ± 32	26.4–(> 33)^83^^,d^	5.4–34.6^c^	770	Gush Halav, Israel (33° 02′ N, 35° 27′ E)	Subtropics	791	16.7	1.3	28.2

Numbers in parentheses after the species names represent sample sizes.

TNZ, thermoneutral zone; AP, annual precipitation; AMT, annual mean temperature; MT, minimal temperature; TAR, temperature annual range (climatic parameters were downloaded from the Worldclim database^49^; due to the unknown locality of *S. cyanus*, climatic data from the whole species range were used).

^a^J. Okrouhlík, unpublished data, soil temperature 30 cm deep, yearly range in 2017/2018, measured every hour.

^b^J. Okrouhlík, unpublished data, soil temperature 10 cm deep, yearly range in 2017/2018, measured every hour.

^c^M. Lövy, unpublished data, soil temperature 20 cm deep, yearly range in 2014/2015, measured every 15 min.

^d^Upper limit is not known, but our unpublished data indicate that it is above 33 °C in *N. galili*.

^e^Winter season.

- Smaller T_diff_ will be found on the venter compared with the dorsum and feet at lower T_a_s confirming the existence of the ventral heat dissipation surface in all species. At these low T_a_s, larger T_diff_ on the feet and dorsum is a consequence of vasoconstriction in the feet, and better fur insulation of the dorsum. Furthermore, T_diff_ will be smaller in all three body regions at the highest T_a_s when body heat needs to be dissipated at the highest possible rate from all body surfaces to avoid overheating.
- T_diff_ will be generally smaller on the venter and dorsum in social compared to solitary species indicating easier heat dissipation through these regions in social species. No differences in T_diff_ are expected for the feet because they are less haired in both solitary and social species.
- The pattern of T_diff_ along the ventral body region will be homogeneous in social species, and heterogeneous in solitary species. The heterogeneity in T_diff_ in solitary species will be caused by smaller T_diff_ on the less furred chest compared to higher T_diff_ of other areas of the venter. Next, the venter will be homogeneous in T_diff_ in all species at highest T_a_s, when body heat needs to be dissipated by the whole ventral surface to avoid overheating.

## Material and methods

### Tested animals

Altogether 73 individuals from seven species of subterranean rodents differing in body mass, phylogenetic relatedness, and sociality were studied (Table 1). All animals were adult non-breeders, or their breeding history was unknown in solitary species, but none of them showed signs of recent breeding, which may theoretically influence measured parameters. For the purpose of this study, we used the following taxa. African mole-rats (Bathyergidae): the social Ansell’s mole-rat *Fukomys anselli* (Burda, Zima, Scharff, Macholán & Kawalika 1999) occupies the miombo in a small area near Zambia’s capital Lusaka; another social species of the genus *Fukomys* is named here as *Fukomys* “Nsanje” because founders of the breeding colony were captured near town Nsanje in south Malawi. Although we used name *Fukomys darlingi* (Thomas 1895) for mole-rats from this population in previous studies (e.g.^38,49^), its taxonomic status is still not resolved; the social common mole-rat *Cryptomys hottentotus hottentotus* (Lesson, 1826) occurs in mesic and semi-arid regions of southern Africa; the solitary Cape dune mole-rat *Bathyergus suillus* (Schreber, 1782) inhabits sandy soils along the south-western coast of South Africa; and the solitary Cape mole-rat *Georychus capensis* (Pallas, 1778) occupies mesic areas of the South Africa^50^. In addition, we studied the social coruro *Spalacopus cyanus* (Molina, 1782) (Octodontidae) occupying various habitats in Chile^51^; and the solitary Upper Galilee Mountains blind mole rat *Nannospalax galili* (Nevo, Ivanitskaya & Beiles 2001) (Spalacidae) from Israel^52^. Further information about the species including number of individuals used in the study, their physiology and ecology is shown in Table 1.

All experiments were done on captive animals. *Georychus capensis*, *C. hottentotus*, and *B. suillus*, were captured about four months before the experiment, and kept in the animal facility at the University of Pretoria, South Africa (temperature: 23 °C; humidity: 40–60%, photoperiod: 12L:12D). The animals were housed in plastic boxes with wood shavings used as a bedding. *Cryptomys hottentotus* and *G. capensis* were fed with sweet potatoes; *B. suillus* with sweet potatoes, carrots, and fresh grass. *Fukomys anselli*, *F.* “Nsanje”, *N. galili*, and *S. cyanus* were kept for at least three years in captivity (or born in captivity) before the experiment in the animal facility at the University of South Bohemia in České Budějovice, Czech Republic (temperature: African mole-rats 25 °C, *N. galili* and *S. cyanus* 23 °C; humidity: 40–50%, photoperiod: 12L:12D). The animals were kept in terraria with peat as a substrate and fed with carrots, potatoes, sweet potatoes, beetroot, apple, and rodent dry food mix ad libitum.

### Experimental design

We measured T_b_ and T_s_ in all species at six T_a_s (10, 15, 20, 25, 30 and 35 °C). Each individual of all species was measured only once in each T_a_. Measurements were conducted in temperature controlled experimental rooms in České Budějovice and Pretoria. Each animal was tested on two experimental days.

The animals were placed in the experimental room individually in plastic buckets with wood shavings as bedding. On the first day, the experimental procedure started at T_a_ 25 °C. They spent 60 min of initial habituation in the first T_a_ after which T_b_ and T_s_ were measured as described in the following paragraphs. The T_a_ was then increased to 30 °C and 35 °C, respectively. After the experimental room reached the focal T_a_, the animals were left minimally 30 min in each T_a_ to acclimate, and the measurements were repeated. Considering their relatively small body size, tested animals were very likely in thermal equilibrium after this period because mammals of a comparable body mass are thermally equilibrated after similar period of acclimation^53–56^. On the second day, the procedure was repeated with the initial T_a_ 20 °C and decreasing to 15 °C and 10 °C, respectively. The time span between the measurements of the same individual in different T_a_ was at least 150 min. Between experimental days, the animals were kept at 25 °C in the experimental room (individuals of social species were housed together with their family members).

### Body temperature measurements

We used two sets of equipment to measure animal T_b_ and T_s_. In *B. suillus*, *G. capensis*, and *C. hottentotus*, T_b_ was measured by intraperitoneally injected PIT tags (< 1 g, LifeChip with Bio-Thermo Technology; Destron Fearing Corp., Dallas, Texas, USA, accuracy 0.5 °C, resolution 0.1 °C). A vet injected the tags under anaesthesia (Isoflurane) three months before the experiment. The tags were calibrated by the manufacturer and were read using a Global Pocker Reader EX (Destron Fearing Corp., Dallas, Texas, USA). T_s_ was measured by FLIR SC325 thermal camera (FLIR Systems, Inc., Wilsonwille, Oregon, USA; sensitivity < 50 mK, accuracy ± 2%, frame rate 31 fps, calibrated by the manufacturer).

Core body temperatures of *F.* “Nsanje”, *F. anselli*, *N. galili*, and *S. cyanus* were measured using a RET-2 temperature probe (Physitemp Instruments LLC., Clifton, New Jersey, USA; tip diameter 3.2 mm, inserted at least 2 cm in the rectum, accuracy 0.1 °C) connected to Thermalert TH-8 (Physitemp Instruments LLC., Clifton, New Jersey, USA; resolution 0.1 °C). This procedure took less than 30 s. The apparatus was verified against a thermometer (EL-USB-2-LCD+; Lascar Electronics Ltd., Salisbury, UK; overall accuracy 0.45 °C) calibrated by an accredited laboratory. Both means of measuring T_b_ have been shown to provide almost identical results in small mammals including one species belonging to African mole-rats^57–59^. Surface temperature was measured using a Workswell WIRIS thermal imaging system (ver. 1.5.0, Workswell s.r.o., Praha, Czech Republic, sensitivity 30 mK, accuracy ± 2%, frame rate 9 fps, calibrated by the manufacturer). Both thermal cameras were calibrated prior to measurement of each animal, and we used the same software to process the raw thermograms (see below).

To measure T_s_, a focused radiometric video of the animal was taken with its different body parts exposed perpendicularly to the camera. More specifically, the animals were held hanging by the loose skin around the tail, and their dorsal and ventral body regions were sequentially exposed to the camera. This procedure took less than 30 s. To ensure unbiased T_s_ measurements, all fans in the experimental room were switched off during measurements, and a non-uniformity correction (type of calibration of the camera) was performed just before measuring each animal. Since the seven tested species were of different sizes, and the lenses of the two thermal cameras had different focal lengths, and thus field of views, the animals were filmed at different distances from the camera to fully utilise the resolution of the two cameras. Distances of animals to the camera lens were 38–42 cm for *N. galili*, *F. anselli*, *F.* “Nsanje”, and *S. cyanus*, 53–57 cm for *C. hottentotus*, 64–68 cm for *G. capensis*, and 86–92 cm for *B. suillus*. Room air temperature and humidity were monitored throughout the trials (EL-USB-2-LCD+; Lascar Electronics Ltd., Salisbury, UK; calibrated by a certified laboratory). Humidity in both labs ranged from 47 to 72% during all experiments.

To assess the heat dissipation (which is related mainly to insulative properties of the integument) of each species regardless of their actual value of T_b_, we introduced T_diff_ parameter defined as the difference between T_b_ and T_s_ of a particular body region of each individual (the dorsum, the venter, and the feet) at each T_a_.

Handling stress may influence T_b_ and may also evoke vasoconstriction on the periphery and thus potentially affect T_s_^58^. To test the potential influence of handling procedures employed in our study on T_s_ and T_b_, we carried out several simple experiments. Firstly, we measured T_b_ of three individuals of *F. mechowii* (species not included in the present study, but in^31^) by intraperitoneal probes (G2-HR E-Mitter, Starr Life Sciences Corp., Oakmont, Pennsylvania, USA; the only species with these probes in our breeds), and found no significant differences in T_b_ prior to or after the 30 s handling period (T_b_ was 34.0 ± 0.6 °C and 34.1 ± 0.7 °C before and after handling, respectively; paired t-test: t = −0.42, df = 3, *p* = 0.70). Secondly, to exclude the possibility of a long-term effect of repeated handling on T_b_, we simulated the measurement procedure with another three individuals of the same species with intraperitoneally injected PIT tags obtaining T_b_s without direct contact. We placed mole-rats singly in a bucket and after 150 min we measured their T_b_. Subsequently, we lifted them for a period of one min and returned them into the bucket. After 150 min, we again measured their T_b_s. Lifting of the mole-rats and temperature measurement was repeated once again (Note that 150 min is the minimal period between two manipulations of each individual in our experiment). The T_b_s did not change during these three subsequent measurements (Generalized Least Squares model [GLS]: F = 0.7, *p* = 0.544; T_b_ values were 33.14 ± 0.6, 33.13 ± 0.77 and 33.23 ± 0.92 °C). Thirdly, to rule out a possible effect of handling on T_s_, we measured dorsal T_s_ in ten individuals of *F.* “Nsanje” (species included in the present study) before and after the handling period which took usually less than 30 s. Similarly, we did not find significant differences (T_s_ was 30.5 ± 1.1 °C and 30.3 ± 1.1 °C before and after the 30 s handling, respectively; paired t-test: t = 1.78, *df* = 9, *p* = 0.11). It should be noted that all animals are accustomed to this handling, as they undertake it on a weekly basis during routine activities, such as weighing, cleaning of terraria, and miscellaneous behavioural and physiological experiments. We therefore suggest that the different approaches in obtaining T_b_, and different time in captivity for experimental animals did not affect our results substantially.

### Thermogram processing and analysis

The thermographic camera produces (sequences of) images in the infrared range, so-called thermograms. Sharp and high contrast radiometric thermograms of the dorsum, the venter and one hind foot as a representative of the feet, were captured from the raw radiometric video in the software CorePlayer (ver. 1.7.70.320; Workswell s.r.o., Praha, Czech Republic; https://workswell-thermal-camera.com/workswellcoreplayer), and processed into non-radiometric thermograms by specifying temperature calculation parameters in the software ThermoFormat (Workswell s.r.o., Praha, Czech Republic; https://workswell-thermal-camera.com/workswell-thermoformat). These parameters were entered as follows: fur emittance was set to 0.97 (e.g.^60^); air humidity, air temperature, and reflected temperature were entered as means of experimental room humidity and air temperature during the course of T_s_ measurement of each species at given T_a_; distance was set as the distance of camera lens from the animal. Although Šumbera et al.^31^ identified a few other body surface areas through which body heat is mainly passively dissipated in mole-rats (peripalpebral, nose, and ear area), we did not include them in our study due to their relatively small area, and thus very low contribution to overall heat dissipation.

**Figure 1:**
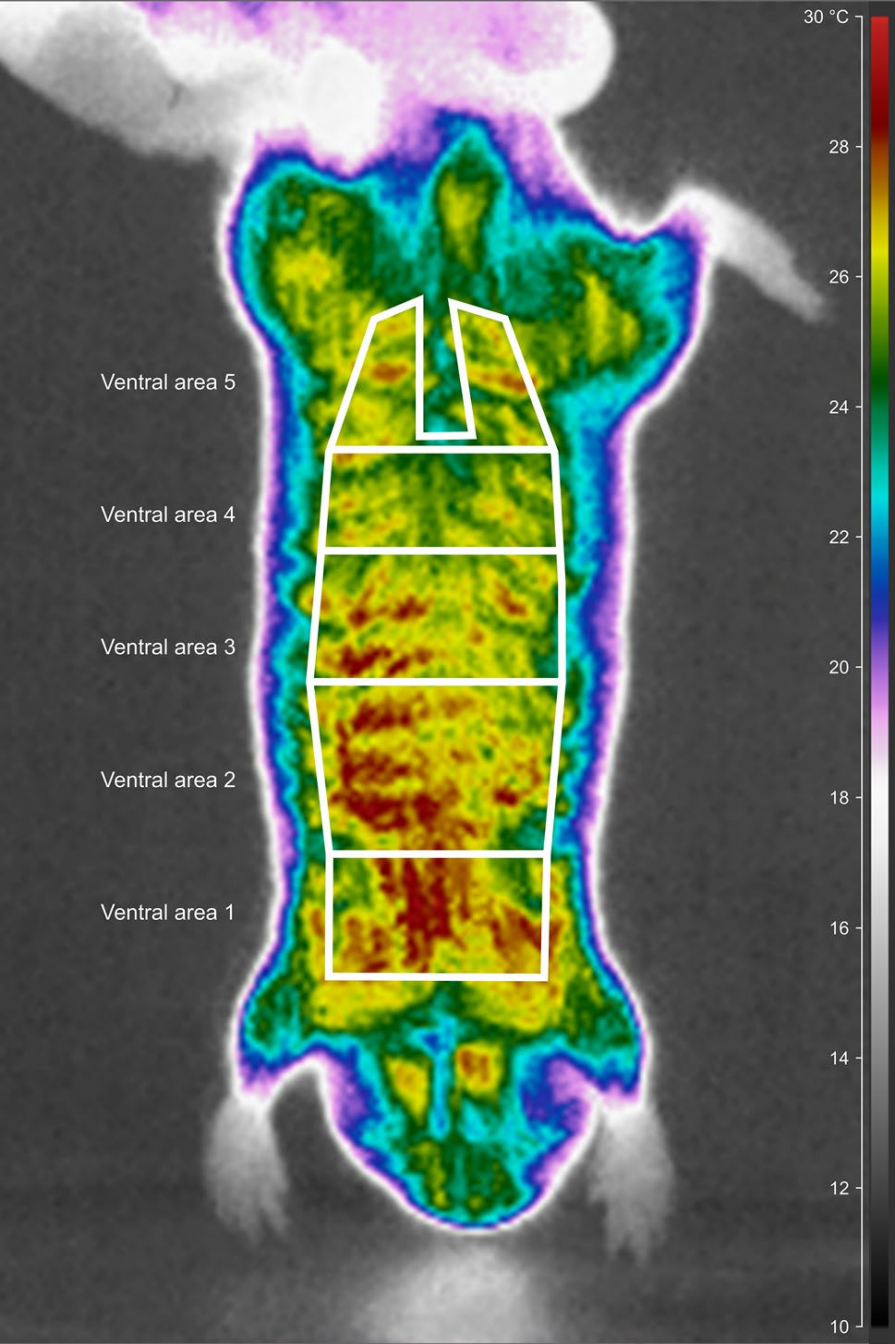
A schematic illustration of the layout of the five ventral areas for which surface temperatures were measured. The background is an infrared thermogram of the Ansell’s mole-rat, *F. anselli* taken after acclimation at 10 °C. The figure was prepared using the program Inkscape 0.92 (https://inkscape.org/).

Processed thermograms were used to infer mean T_s_ of the whole ventral and dorsal body region, and the feet necessary for the calculation of T_diff_. The analysed body regions were manually marked out with a polygonal region of interest in the CorePlayer (see Fig. 1 as an example for polygon of the whole venter). The region of interest was adjusted to fit onto the part of the body region of the particular individual perpendicularly oriented to the camera lens. Additionally, the venter was divided in the anterior–posterior axis into five areas (Fig. 1: 1—between the front legs, 2—to the widest part of the chest, 3—to the end of the rib cage, 4—to the hips, and 5—between hind legs without the anogenital area).

### Data analyses

In all analyses performed in this study, T_diff_ was used as a dependent variable assuming Gaussian error distribution. In all models described below, body region (the dorsum, the venter, and feet), social organisation (social and solitary), and ventral area (1–5) were treated as explanatory categorical variables, whereas T_a_ was treated as a covariate. Means ± SD are given throughout the text. The sex of the tested animals was not included as an explanatory variable, mainly because of a relatively low number of males and females tested. Nevertheless, we did not expect any differences because there are no remarkable sex differences in thermal biology of subterranean rodents apart from body mass if females are not pregnant or lactating pups^25^. To test our hypotheses, we fitted the models on subsets of data divided according to body region and selected T_a_ values. All analyses were carried out in R^61^ and all figures were edited using the program Inkscape 0.92 (https://inkscape.org/).

### T_b_, T_s_, and T_diff_ over the T_a_ gradient

Prior to testing of our hypotheses, we performed the following basic statistical exploratory analyses of T_b_ and T_s_. For each species, we firstly calculated a piecewise linear regression implemented in the R package *Segmented*^62^ to assess whether there is a breakpoint T_a_, at which the regression curve characterising the change in T_b_ (expressed as mean values for each T_a_ for each species) for a given species changes its slope. Secondly, we calculated a linear regression to characterise a change in T_s_ (expressed as mean values for each T_a_ for each species) in response to increasing T_a_. We used Bonferroni procedure to correct the α of the tests since we calculated one piecewise linear regression for T_b_ for each of the seven species (adjusted α = 0.0071) and 21 linear regressions for T_s_ (adjusted α = 0.0024).

For each species separately, we tested whether T_diff_ differs amongst the three body regions, i.e. the dorsum, the venter, and the feet, using GLS marginal models implemented in the *nlme* package^63^. One GLS model was calculated for each of three T_a_s − 10 and 35 °C as extremes in order to obtain information about T_diff_ in cold and hot conditions, respectively, and 30 °C which represents TNZ of all the studied species (Table 1). In all GLS models, the body region was the explanatory variable, and the identity of tested individuals was included to avoid pseudo-replication. We used the Bonferroni procedure to correct the significance level of the tests because we tested each T_a_ separately for seven species and three T_a_s (adjusted α = 0.0024), and then performed a similar post-hoc comparison 13-times (adjusted α = 0.0038).

For each species separately, we first calculated three linear regressions to test whether the slope characterising the change of T_diff_ in response to increasing T_a_ for each of the three body regions is different from zero. For each body region, T_diff_ value for a given T_a_ was calculated as the mean obtained from all individuals within a given species, for which we obtained a complete set of T_s_ values from all three body regions at all six T_a_s. Afterwards, a homogeneity-of-slopes model was calculated to test whether these three regression lines are parallel to each other, i.e. there is no interaction between body region and T_a_. The function “emtrends” implemented in the R package *emmeans*^64^ was used to calculate post-hoc comparisons to find differences between the slopes for the dorsum, the venter, and the feet in these models. We used the Bonferroni procedure to correct the significance level of the tests we performed for each of the seven species: (a) three similar regression models (adjusted α = 0.0024) and (b) altogether seven homogeneity-of-slope models with a post-hoc test (adjusted α = 0.0071).

### T_diff_ of the three body regions in solitary and social species

To assess the effect of the social organization on T_diff_ (expressed as a mean value for each T_a_ for each species) in each of the three body regions, we fitted Generalized Linear Mixed Models in a Bayesian framework using Markov Chain Monte Carlo sampling algorithm (MCMC) implemented in the R package *MCMCglmm*^65^. This approach was used to account for non-independence in T_diff_ measurements arising due to shared phylogenetic ancestry, and therefore a random effect of phylogeny was included in all models. We controlled for phylogenetic relatedness using the subset tree of the mammal phylogeny by Upham et al.^66^. The set of subtrees was retrieved via an online tool: vertlife.org/phylosubsets/. The maximum credibility tree was then inferred using the R package *phangorn*^67^, and taxa labels were changed using the R package *phytools*^68^ in order to merge the taxa with the comparative dataset.

Parameter estimates for fixed and random effects of each model were obtained from sampling posterior distributions by running 2,500,000 MCMC iterations with a burn-in period of 20% and a thinning interval sampling each 1000th iteration. Estimates of each parameter are thus based on 2000 samples from a posterior distribution. In all models, we used weakly informative priors for variance components (V = 1, ν = 0.02), and a default prior specification for fixed effects, i.e. mean centred on zero with a very large variance (µ = 0, V = 10^8^), to let the posterior be determined mostly by the information in the data. Mixing and convergence of MCMC chains were assessed by visual inspection of both time-series and density plots, as well as by calculating autocorrelation among successive MCMC samples. There was no apparent trend among MCMC samples in time-series plots, and autocorrelation was low in each fitted model. Effective sample size was ~ 2000 for all estimated parameters in all models, suggesting generally good mixing and convergence properties of MCMC chains.

For each of the three body regions separately, we fitted a model of the formula: T_diff_ ~ T_a_ + social organisation + T_a_: social organisation (the same T_diff_ datasets as for linear regressions were used for these models). Beside the random effect of the phylogeny accounting for evolutionary history of the studied taxa, we included also a random effect of species to account for species-specific effects on the variability in T_diff_ in response to T_a_. This model allowed us to test whether the slopes for the change in T_diff_ as a response to increasing T_a_ differ between solitary and social species. Interaction term was considered only when its effect in a model was significant (see highest posterior density (HPD) intervals and/or pMCMC in Table 3), and the deviance information criterion (DIC) indicated a better fit (lower DIC) of the model with than without interaction. If the effect of interaction was not significant, we ran the model without the interaction term, and interpreted only the effects of T_a_ and social organization, respectively.

### T_diff_ along the venter in solitary and social species

For each species individually, we tested whether T_diff_ differs among the five ventral areas using GLS models. One GLS model was calculated for each T_a_ of 10, 30 and 35 °C. In all GLS models, the ventral area was the explanatory variable (a factor with five levels: ventral area 1—ventral area 5, see Fig. 1). We used the Bonferroni procedure to correct the significance level of the tests since we tested each of three T_a_s separately for seven species (adjusted α = 0.0024). In all GLS models, an identity of tested individuals was included to avoid pseudo-replication.

In addition, for each of the three T_a_, we fitted one MCMCglmm of the form: T_diff_ ~ ventral area + social organisation + ventral area: social organisation, with random effects of the phylogenetic relatedness and species. This model allowed us to test whether the pattern of mean change in T_diff_ (calculated as the mean from all individuals of each species entering GLS analysis mentioned above) across five ventral areas differed between solitary and social species. The effect of interaction term was considered significant if the HPD intervals did not include zero (see HPD intervals and/or pMCMC in Table 4), and DIC of the model with interaction was lower than that of the model without it.

### Ethical approval

The experimental procedures in the Czech Republic were approved by the University of South Bohemia Animal Welfare committee and Ministry of Education, Youth and Sports of the Czech Republic (Permission no. MSMT-26065/2014-12). The experimental procedures in South Africa were approved by University of Pretoria Ethics Committee (Permission no. EC069-16). All procedures were performed in accordance with relevant guidelines and regulations.

## Results

### T_b_, T_s_, and T_diff_ over the T_a_ gradient

In all species apart from *C. hottentotus* and *S. cyanus*, T_b_ was stable from 10 up to 25 °C (the slopes of segmented regressions were close to 0), showing breaking points between 26.6 and 28.8 °C, after which T_b_ started to increase (see Table 2 for details). For *C. hottentotus*, T_b_ increased across the whole T_a_ range (the slope was significantly different from 0 for T_b_ values before the breaking point), but there was a relatively steeper increase between 30 and 35 °C (breaking point at 29.3 ± 0.2 °C, Table 2). For *S. cyanus*, T_b_ was stable only from 10 to 20 °C, with a breaking point at T_a_ of 23.1 ± 1.1 °C.

**Table 2:** Results of piecewise linear regressions characterising a change in core body temperature (T_b_) and linear regressions characterising a change in the surface temperature (T_s_) for three body regions in response to increasing ambient temperature (T_a_) in seven subterranean rodent species. Breaking points and slopes are provided as mean ± SEM; *p* values for the slopes before the breaking point are in parentheses; statistically significant tests after the Bonferroni procedure α = 0.0071 for T_b_ and α = 0.0024 for T_s_ are marked with asterisks.

Species	T_a_ breaking point for T_b_ (°C)	Slope before the breaking point of T_b_ (*p* value)	Slope after the breaking point of T_b_	T_s_ Dorsum (F and *p* value)	T_s_ Venter (F and *p* value)	T_s_ Feet (F and *p* value)
*B. suillus*	27.5 ± 0.9	0.052 ± 0.010 (0.035)	0.244 ± 0.033	609.6, 1.6 × 10^–5^*	850.9, 8.2 × 10^–6^*	125.5, 3.6 × 10^–4^*
*G. capensis*	28.6 ± 1.4	0.048 ± 0.020 (0.137)	0.264 ± 0.066	386.6, 3.9 × 10^–5^*	157.5, 2.3 × 10^–4^*	216.1, 1.2 × 10^–4^*
*C. hottentotus*	29.3 ± 0.2	0.069 ± 0.005 (0.004)*	0.397 ± 0.015	615.7, 1.6 × 10^–5^*	263.6, 8.4 × 10^–5^*	56.1, 0.0017*
*F. anselli*	28.8 ± 0.9	0.072 ± 0.019 (0.064)	0.361 ± 0.067	137.3, 3.0 × 10^–4^*	74.1, 0.001*	160.0, 2.3 × 10^–4^*
*F.* “Nsanje”	26.8 ± 0.5	− 0.030 ± 0.012 (0.131)	0.054 ± 0.040	309.1, 6.1 × 10^–5^*	52.1, 0.002*	1229.7, 3.9 × 10^–6^*
*S. cyanus*	23.1 ± 1.1	0.002 ± 0.025 (0.942)	0.274 ± 0.035	1060.3, 5.3 × 10^–6^*	670.9, 1.3 × 10^–5^*	350.4, 4.8 × 10^–5^*
*N. galili*	26.6 ± 1.6	0.059 ± 0.013 (0.047)	0.192 ± 0.044	1036.3, 5.5 × 10^–6^*	921.5, 7.0 × 10^–6^*	382.4, 4.0 × 10^–5^*

**Figure 2:**
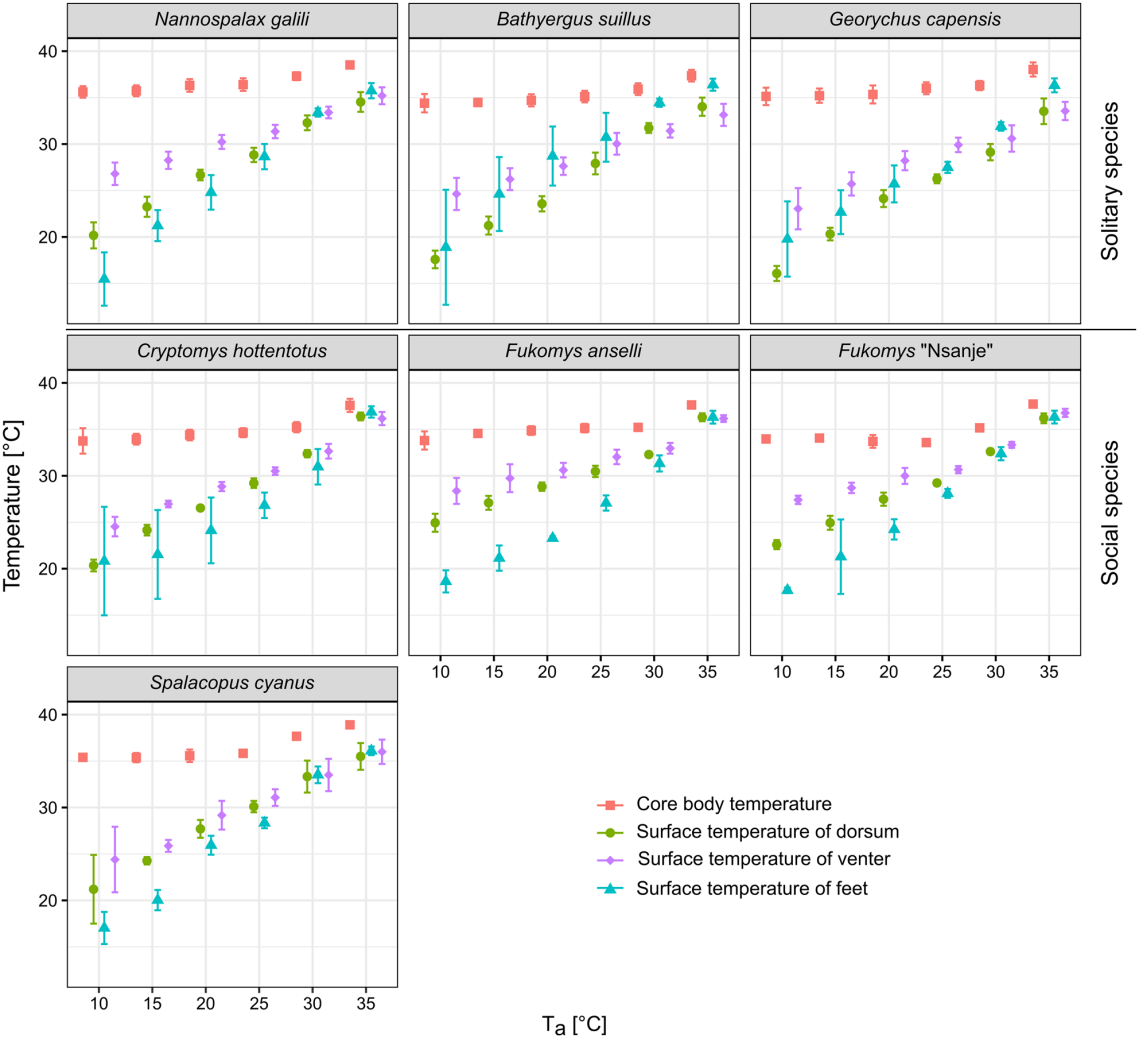
Core body temperatures and surface temperatures of the dorsum, the venter, and the feet measured across the range of ambient temperatures in seven subterranean rodent species. Means ± SD are depicted. The figure was prepared using the program Inkscape 0.92 (https://inkscape.org/).

Surface temperature for the dorsum, the venter, and the feet increased in all species over the range of T_a_ (Linear regressions, all *p* < 0.002 were significant after the Bonferroni procedure, Fig. 2 and Table 2). In contrast to T_b_, T_s_ of all three regions showed markedly higher differences among the species, with the highest value for the venter at lower T_a_s, but these differences became smaller at 30 and 35 °C (Fig. 2).

For each of the seven species, T_diff_ differed among the three body regions at 10 °C (*p* < 0.0001, Supplementary Table S2). In *N. galili*, *F. anselli*, *F.* “Nsanje”*,* and *S. cyanus*, T_diff_ was the lowest for the venter, and the highest for the feet, with the dorsum having intermediate T_diff_ significantly different from both (*p* < 0.001, for all pairwise comparisons see Supplementary Table S2). In *B. suillus*, *G. capensis*, and *C. hottentotus*, T_diff_ was lower only for the venter than the dorsum (*p* < 0.001), and the feet did not differ from either of the two.

At 30 °C, T_diff_ differed among the three body regions in *N. galili*, *B. suillus,* and *F. anselli* only (*p* < 0.0001, Supplementary Table S2). While both the venter and the feet had significantly lower T_diff_ than the dorsum in *N. galili* (*p* < 0.001), T_diff_ was lower for the feet than for both the venter and the dorsum in *B. suillus* (*p* < 0.001). In *F. anselli*, the ventral T_diff_ was lower than the dorsal one, with T_diff_ of the feet being the highest (*p* < 0.001).

At 35 °C, T_diff_ differed among the three body regions only in *N. galili*, *B. suillus,* and *G. capensis* (*p* < 0.0001, Supplementary Table S2). The feet of *N. galili* had lower T_diff_ than the venter and the dorsum, with the latter having the highest T_diff_ (*p* < 0.001). In both *B. suillus* and *G. capensis*, the feet had lower T_diff_ than both the dorsum and the venter (*p* < 0.001) which had similar T_diff_.

**Figure 3:**
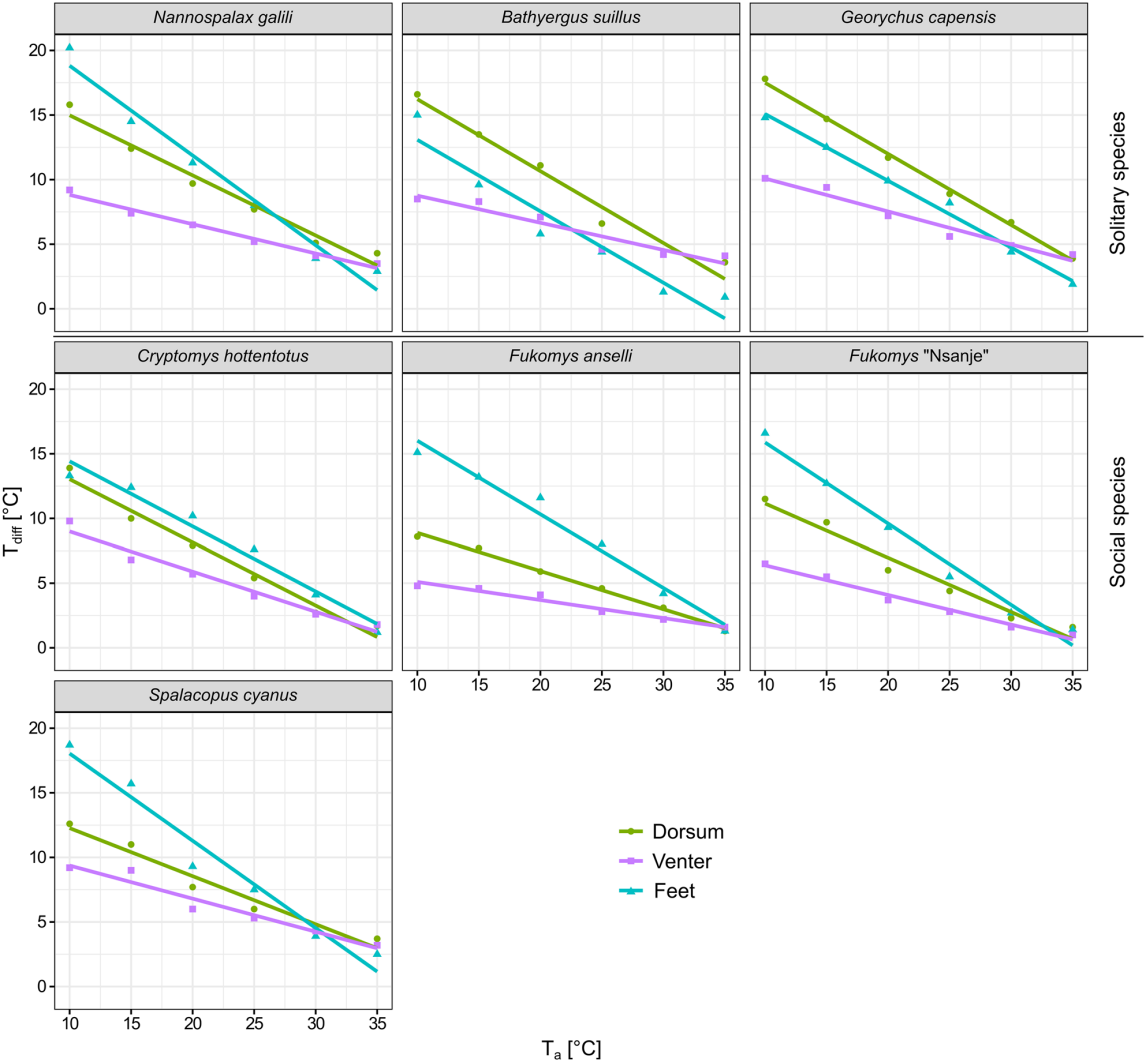
The difference between core body temperature and surface temperature (T_diff_) for the dorsum, the venter, and the feet across the range of ambient temperature (T_a_) in seven subterranean rodent species. Points indicate mean values, straight lines are the regression lines predicted by linear models. The figure was prepared using the program Inkscape 0.92 (https://inkscape.org/).

For each of the seven species, T_diff_ decreased with increasing T_a_ in all three body regions (see Table 3 for statistical details and Fig. 3). Among the three body regions, the ventral T_diff_ showed the smallest change in response to increasing T_a_ (i.e. a less steep slope), and it differed significantly from those of both the dorsum and the feet in five species. In *S. cyanus*, it differed from the feet only, and in *C. hottentotus*, no differences among all three respective slopes were found. Besides, the slopes for the dorsum were significantly steeper than slopes for the feet in *F. anselli*, *F.* “Nsanje”, *S. cyanus*, and *N. galili*.

**Table 3:** Results of the linear regressions testing whether the slopes characterising a decrease in the change of the difference between core body and surface temperatures (T_diff_) in response to increasing ambient temperatures (T_a_) differ from zero for the dorsum (D), venter (V) and feet (F) and of the homogeneity-of-slope models (Body region × T_a_) testing whether these slopes differ among the three body regions in seven subterranean rodent species. The degrees of freedom were 1 and 4 for the linear regressions and 2 and 12 for the homogeneity-of-slope models; the goodness of fit of the regression is presented by the adjusted R^2^; the *p* value of post-hoc pairwise comparisons for the homogeneity-of-slope models are shown in the columns V vs. D, V vs. F, and D vs. F; statistically significant results after Bonferroni correction α = 0.0024^a^ or α = 0.0071^b^ applied are marked with an asterisk.

Species	Results of linear regressions testing non-zero slopes			Results of homogeneity-of-slope models			
	Dorsum (R^2^ adj)^a^	Venter (R^2^ adj)^a^	Feet (R^2^ adj)^a^	Body region × T_a_^b^	*p* value of post-hoc comparison		
					V versus D^a^	V versus F^a^	D versus F^a^
*B. suillus*	t = −11.1, *p* < 0.001* (0.96)	t = −6.1, *p* < 0.001* (0.88)	t = −7.1, *p* = 0.002* (0.91)	F = 12.1, *p* = 0.001*	0.003*	0.003*	0.999
*G. capensis*	t = −38.0, *p* < 0.001* (1.00)	t = −10.0, *p* < 0.001* (0.95)	t = −21.4, *p* < 0.001* (0.99)	F = 54.2, *p* < 0.001*	< 0.001*	< 0.001*	0.552
*C. hottentotus*	t = −13.3, *p* < 0.001* (0.97)	t = −10.4, *p* < 0.001* (0.96)	t = −11.8, *p* < 0.001* (0.97)	F = 8.5, *p* = 0.005*	0.013*	0.008*	0.956
*F. anselli*	t = −25.0, *p* < 0.001* (0.99)	t = −10.0, *p* < 0.001* (0.95)	t = −13.4, *p* < 0.001* (0.97)	F = 66.2, *p* < 0.001*	0.004*	< 0.001*	< 0.001*
*F.* “Nsanje”	t = −10.6, *p* < 0.001* (0.96)	t = −14.9, *p* < 0.001* (0.98)	t = −14.2, *p* < 0.001* (0.98)	F = 31.7, *p* < 0.001*	0.007*	< 0.001*	0.003*
*S. cyanus*	t = −10.5, *p* < 0.001* (0.96)	t = −8.4, *p* = 0.001* (0.93)	t = −10.1, *p* < 0.001* (0.95)	F = 21.1, *p* < 0.001*	0.231	< 0.001*	0.002*
*N. galili*	t = −12.4, *p* < 0.001* (0.97)	t = −14.4, *p* < 0.001* (0.98)	t = −11.6, *p* < 0.001* (0.96)	F = 31.4, *p* < 0.001*	0.004*	< 0.001*	0.006*

### T_diff_ of the three body regions in solitary and social species

**Figure 4:**
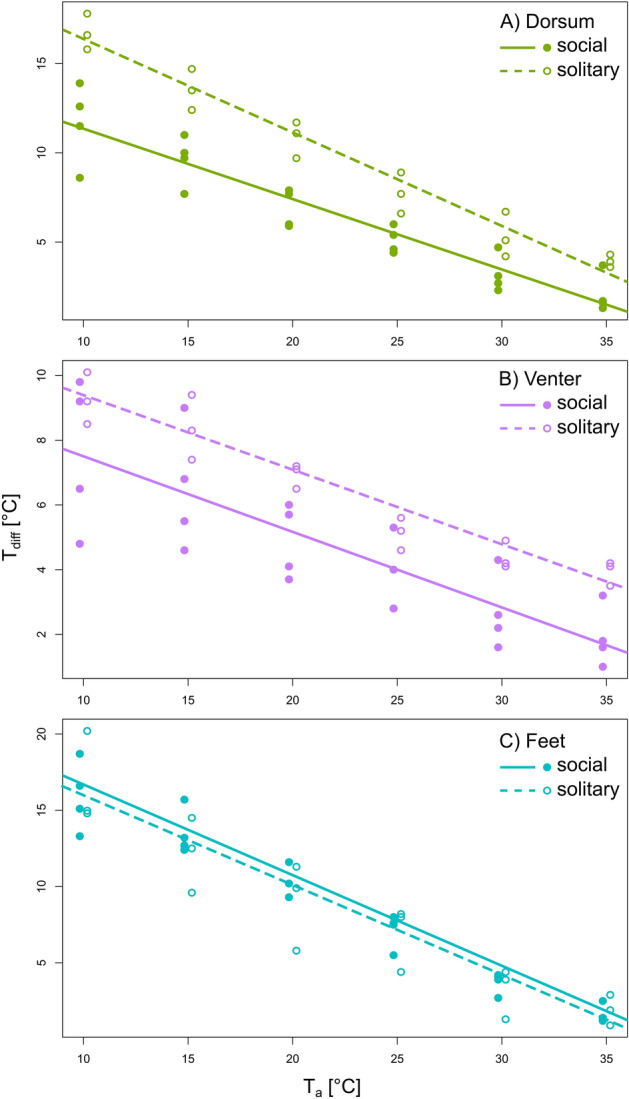
The difference between core body temperature and surface temperature (T_diff_) in response to increasing ambient temperature (T_a_) for the dorsum (**A**), the venter (**B**), and the feet (**C**) between solitary and social species. Lines are the regression lines predicted by MCMCglmm. The figure was prepared using the program Inkscape 0.92 (https://inkscape.org/).

The comparison among the slopes of regression lines characterising the response of T_diff_ to increasing T_a_ for the dorsum, the venter, and the feet differed between solitary and social species (Fig. 4). For the dorsum, T_diff_ was significantly higher in solitary than in social species across the whole T_a_ range (pMCMC < 0.0001), and it decreased relatively more in solitary (slope − 0.52) than in social species (slope − 0.39) for a given increase in T_a_ (Fig. 4A and Table 4, DIC = 134.4 and 119.8 for models without and with the interaction between T_a_ and social organisation, respectively). For the venter, the slopes characterising the change in T_diff_ in response to increasing T_a_ did not differ between solitary and social species (slope − 0.23, DIC = 93.4 and 95.6 for models without and with the interaction between T_a_ and social organisation, respectively; pMCMC = 0.884), but there was a tendency (pMCMC = 0.079) for higher T_diff_ in solitary compared with social species (Fig. 4B and Table 4). For the feet, T_diff_ did not differ between solitary and social species across the T_a_ range (slope − 0.59, Fig. 4C, and Table 4, DIC = 145.8 and 148.1 for models without and with the interaction between T_a_ and social organisation, respectively; pMCMC = 0.902).

**Table 4:** Relationships between the difference between core body and surface temperature (T_diff_) of the three body regions, ambient temperature (T_a_), and social organisation (solitary vs. social) in seven species of subterranean rodents. 2.5% HPD and 97.5% HPD represent lower and upper highest posterior density intervals; pMCMC denotes to the *p* value obtained from MCMCglmm; T_a_ × Social organisation = interaction between T_a_ and social organisation.

Body region	Fixed term	Estimate (β)	2.5% HPD	97.5% HPD	pMCMC
Dorsum	Intercept	15.237	12.534	17.958	
	T_a_	− 0.394	− 0.437	− 0.349	< 0.0001
	Social organisation (solitary)	6.358	3.664	8.995	0.001
	T_a_ × Social organisation	− 0.131	− 0.190	− 0.059	< 0.0001
Venter	Intercept	9.870	6.637	13.007	
	T_a_	− 0.233	− 0.257	− 0.208	< 0.0001
	Social organisation (solitary)	1.962	− 0.124	4.427	0.079
	T_a_ × Social organisation	0.004	− 0.046	0.053	0.884
Feet	Intercept	22.6530	19.3105	26.4544	
	T_a_	− 0.591	− 0.636	− 0.543	< 0.0001
	Social organisation (solitary)	− 0.644	− 3.258	2.197	0.558
	T_a_ × Social organisation	0.005	− 0.092	0.093	0.902

### T_diff_ along the venter in solitary and social species

**Figure 5:**
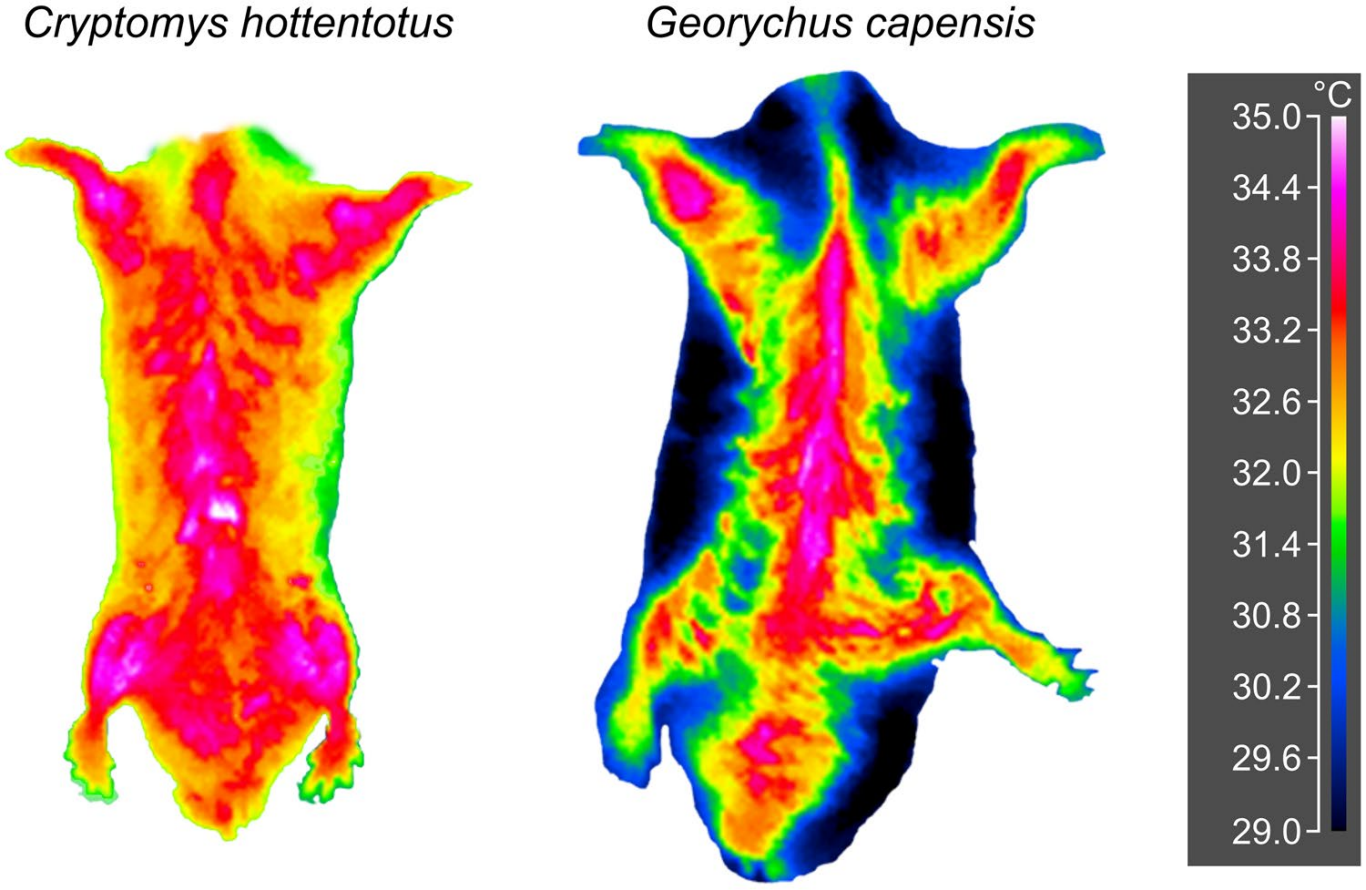
An example of thermogram showing the differences in surface temperature of the venter at 30 °C between the solitary Cape mole-rat *G. capensis* and the social common mole-rat *C. hottentotus*. The figure was prepared using the program Inkscape 0.92 (https://inkscape.org/).

The T_diff_ for the five ventral areas varied for each of the seven species at T_a_ of 10, 30, but not at 35 °C (Table 5; Supplementary Fig. S1; see also Fig. 5 for the comparison of T_s_ pattern of the venter at 30 °C for one solitary and one social mole-rat). In the *N. galili*, *F. anselli*, *F.* “Nsanje”, T_diff_ varied among the five ventral areas at both 10 and 30 °C, whereas for *C. hottentotus* and *S. cyanus,* it differed at 30 °C only. At 35 °C, there were no differences in T_diff_ among the five ventral areas in all species. In solitary *B. suillus* and *G. capensis*, the venter was thermally homogeneous at all T_a_s. There were no significant differences in the pattern of the T_diff_ change among the five ventral areas between solitary and social species in either of the three T_a_s (see Table 6 for statistical details; at 10 °C: DIC = 65.2 and 72.7, at 30 °C: DIC = − 3.6 and 3.1 and at 35 °C: DIC = − 44.1 and − 42.5; the first and second DIC value are for models without and with the interaction between the ventral area and social organisation, respectively).

**Table 5:** The results of GLS models comparing the difference between core body and surface temperature (T_diff_) among the five ventral areas at ambient temperature (T_a_) of 10, 30 and 35 °C in each of the seven subterranean rodent species. For each T_a_, the venter was tested separately; *n* denotes to the number of individuals tested for each T_a_; statistically significant results after Bonferroni correction α = 0.0024 applied are marked with an asterisk; post-hoc comparisons among the areas are depicted by letter combinations in Supplementary Fig. S1.

Species	T_a_ (°C) (*n*)	Fixed term: ventral area
*B. suillus*	10 (10)	F_4, 45_ = 0.7, *p* = 0.616
	30 (9)	F_4, 30_ = 1.3, *p* = 0.302
	35 (10)	F_4, 45_ = 0.4, *p* = 0.817
*G. capensis*	10 (9)	F_4, 40_ = 2.6, *p* = 0.054
	30 (7)	F_4, 30_ = 1.8, *p* = 0.160
	35 (4)	F_4, 15_ = 0.7, *p* = 0.593
*C. hottentotus*	10 (9)	F_4, 40_ = 3.9, *p* = 0.010
	30 (9)	F_4, 40_ = 8.0, *p* < 0.001*
	35 (9)	F_4, 40_ = 4.0, *p* = 0.008
*F. anselli*	10 (8)	F_4, 35_ = 14.7, *p* < 0.001*
	30 (7)	F_4, 30_ = 16.4, *p* < 0.001*
	35 (6)	F_4, 25_ = 3.9, *p* = 0.014
*F.* “Nsanje”	10 (11)	F_4, 50_ = 39.5, *p* < 0.001*
	30 (8)	F_4, 35_ = 11.3, *p* < 0.001*
	35 (6)	F_4, 25_ = 0.4, *p* = 0.814
*S. cyanus*	10 (2)	F_4, 5_ = 0.4, *p* = 0.804
	30 (3)	F_4, 10_ = 43.1, *p* < 0.001*
	35 (5)	F_4, 20_ = 0.7, *p* = 0.586
*N. galili*	10 (17)	F_4, 80_ = 6.1, *p* < 0.001*
	30 (15)	F_4, 70_ = 8.7, *p* < 0.001*
	35 (17)	F_4, 80_ = 1.4, *p* = 0.239

**Table 6:** Relationships between the difference between core body and surface temperature (T_diff_), ventral area (five ventral areas, for details see Fig. 1) and social organisation (solitary vs. social) in seven species of subterranean rodents. 2.5% HPD and 97.5% HPD represent lower and upper highest posterior density intervals; pMCMC denotes to the *p* value obtained from MCMCglmm; ventral area × social organisation = interaction between ventral area and social organisation.

T_a_ (°C)	Fixed terms	Estimate (β)	2.5% HPD	77.5% HPD	pMCMC
10	(Intercept)	7.828	1.728	13.867	0.026
	Ventral area 2	− 0.275	− 1.111	0.456	0.481
	Ventral area 3	− 0.099	− 0.899	0.668	0.817
	Ventral area 4	0.134	− 0.679	0.889	0.743
	Ventral area 5	0.478	− 0.311	1.253	0.225
	Social organisation (solitary)	2.712	− 2.031	6.858	0.187
	Ventral area 2 × social organisation (solitary)	0.122	− 1.109	1.290	0.848
	Ventral area 3 × social organisation (solitary)	0.044	− 1.113	1.141	0.938
	Ventral area 4 × social organisation (solitary)	− 0.354	− 1.526	0.857	0.556
	Ventral area 5 × social organisation (solitary)	− 0.498	− 1.717	0.693	0.375
30	(Intercept)	2.603	0.647	4.388	0.017
	Ventral area 2	− 0.111	− 0.421	0.181	0.461
	Ventral area 3	− 0.119	− 0.415	0.195	0.427
	Ventral area 4	− 0.030	− 0.351	0.253	0.846
	Ventral area 5	− 0.003	− 0.325	0.256	0.980
	Social organisation (solitary)	2.053	0.444	3.786	0.034
	Ventral area 2 × social organisation (solitary)	0.097	− 0.352	0.559	0.657
	Ventral area 3 × social organisation (solitary)	0.049	− 0.390	0.494	0.834
	Ventral area 4 × social organisation (solitary)	− 0.181	− 0.637	0.253	0.409
	Ventral area 5 × social organisation (solitary)	0.092	− 0.340	0.526	0.683
35	(Intercept)	1.441	− 0.949	3.734	0.173
	Ventral area 2	− 0.051	− 0.225	0.103	0.506
	Ventral area 3	− 0.037	− 0.188	0.122	0.641
	Ventral area 4	0.046	− 0.127	0.187	0.543
	Ventral area 5	0.026	− 0.116	0.202	0.728
	Social organisation (solitary)	2.967	1.097	4.553	0.008
	Ventral area 2 × social organisation (solitary)	− 0.048	− 0.275	0.196	0.677
	Ventral area 3 × social organisation (solitary)	− 0.063	− 0.287	0.187	0.585
	Ventral area 4 × social organisation (solitary)	− 0.239	− 0.443	0.016	0.041
	Ventral area 5 × social organisation (solitary)	− 0.166	− 0.406	0.049	0.153

## Discussion

To the best of our knowledge, this study represents the highest number of endotherm species, where heat dissipation has been analysed within a single study using the IRT approach. In several aspects, this allows us to generalise our findings for subterranean rodents. We found that the main heat dissipation areas in all species are present at the venter and the feet. With respect to the effect of sociality, solitary species dissipate less heat through the dorsum than social species and the same tendency was found for the venter. For the feet, there were no differences between both groups. Finally, the pattern of heat dissipation along the venter was not consistent between species and could not be attributed to sociality.

For the purpose of our study, we defined T_diff_ as the difference between T_b_ and T_s_ in each individual and each T_a_ measured. The value of this parameter is predominantly driven by T_s_ which increases remarkably over the gradient of T_a_ for all three body regions with T_b_ being much more stable (Fig. 2). Applying this approach can be important especially in comparative studies using multiple species differing in T_b_ and species-specific change of T_b_ over the gradient of T_a_. This can be illustrated by the results of the segmented analysis when the increase of T_b_ started at 23.1 °C as found in *S. cyanus*, but at 29.3 °C as observed for *C. hottentotus* (Table 2).

Our results demonstrate that the tested body regions play different roles in heat dissipation in subterranean rodents. As predicted (based on^31,34^), at the coldest T_a_ measured (10 °C) we found lower T_diff_ on the venter compared to the dorsum in all studied species (and in *F. anselli*,* F.* “Nsanje”*, S. cyanus*,* N. galili* also compared to the feet) showing relatively higher heat dissipation from this body region (Fig. 3 and Supplementary Table S2). The warmer venter in lower T_a_s compared to other body surfaces was recently also found in three social mole-rat species^35^. This finding corresponds with the pattern found in some other mammals. A venter suitable for heat dissipation can be advantageous as its heat flow may be controlled behaviourally^12,21,31,69^. Although we did not quantify the behaviour of all individuals during the experiments, closing of a ventral thermal window by curling up into a ball was observed in most individuals at lower T_a_s (see Supplementary Fig. S2). Adopting this posture, animals not only minimise heat losses through their venter, but also reduce their effective surface-to-volume ratio bringing additional energetic savings^70^ (note that the same posture is adopted also by torpid small non-volant mammals^71^). On the contrary, at high T_a_s (≥ 30 °C), tested animals usually exposed their venter to the environment mainly by lying on their back with the legs outstretched trying to maximize heat transfer to the surroundings (Supplementary Fig. S2). A large ventral thermal window should thus be advantageous, especially in subterranean mammals because they are predicted to lose the excessive metabolic heat during digging by means of conduction when the venter is in direct contact with the excavated soil and burrow floor^36^.

Our findings also confirm an important role of the feet in thermoregulation of subterranean rodents as has been also shown in two studies on African mole-rats^31,35^. In laboratory rats, the feet together with the distal parts of legs comprise around 10% of the total body surface and serve as an important heat dissipating region^72^. The feet of rodents adapted for digging and removing the soil are larger than in non-fossorial rodents^73,74^, and thus represent a significant proportion of the body surface. The importance of the feet in the thermoregulation of subterranean rodents is likely even higher because of lack of other body appendages, and due to their large surface-to-volume ratio. The feet are usually not well haired which, together with their bare pedal surface, makes them suitable for fast and effective heat transfer. Due to their relatively large size and heat convection via direct contact with the substrate, the feet are far more effective than small areas around the eyes or ears which were also found to lose heat in mole-rats^31^.

In this regard, our results indicate very effective vasoconstriction taking place in the feet to decrease heat losses at the lowest T_a_s. This is supported by the feet being the body region with the lowest T_s_, and thus highest T_diff_ in four of seven tested species at T_a_ of 10 °C (Fig. 2, 3, and Supplementary Table S2). The absence of significant differences between the feet and the other two body regions at this T_a_ in *B. suillus*, *G. capensis*, *C. hottentotus* indicates a very effective insulative ability of their haired body surfaces rather than the absence of effective vasoconstriction in their feet. Importantly, at 35 °C, the feet T_diff_ was significantly smaller than the T_diff_ of other two body regions in three tested solitary species, *N. galili*, *B. suillus*, and *G. capensis* (Fig. 3, Supplementary Table S2). This demonstrates intensive vasodilatation and dissipation of the excessive body heat which cannot be effectively dissipated by other body parts because of the presence of well insulating fur (based on data on fur characteristics for all three species, Vejmělka and Šumbera unpubl. data). Heat dissipation via the feet is likely very relevant, similarly to the venter, especially during digging because of their contact with soil. McGowan et al.^35^ even suggested that the feet with reduced hair coverage are important at high T_a_s when other conductive heat losses are working at their maximal capacity. Since the feet contribute to increased heat dissipation at high T_a_s, but effectively decrease heat loss at low T_a_s, they serve as a typical body-extremity-located thermal window similarly to other mammals (e.g.^3–6^).

The importance of the feet in heat exchange was supported also by behavioural observations during the experiment. At low T_a_s, we observed that many tested animals were sitting on the hind feet with the front feet not in contact with the cold floor (Supplementary Fig. S2). It was also documented that several individuals of *H. argenteocinereus* had some of their feet surprisingly warm while shivering at 10 °C^31^. This indicates that the feet may play an active role in dissipation of excessive heat produced by shivering as suggested by authors, or alternatively, occasional warming of the feet could be related to protection against cold damage by repeated short-term redirection of blood flow into peripheral colder body parts, as showed across diverse mammalian taxa including foxes, rats, or humans (e.g.^4,75,76^).

We demonstrated that tested species and probably other subterranean rodents effectively combine heat dissipation through the venter and the feet, yet each of these body regions acts as a different type of thermal exchanger. The first type emits heat more or less continuously (as indicated by constantly relatively low T_diff_, and by its less steep slope over the gradient of T_a_ compared to the other two body regions; see Fig. 3), and is represented by being less furred, and thus less thermally insulated venter^31^. When necessary in cold conditions, heat dissipation through this surface can be reduced behaviourally by changing the body posture (see above). The second type is represented by the feet with the steeper slope of the T_diff_ change. In the cold temperatures, heat loss may be prevented effectively as for instance indicated in gerbils^77^. In this regard, the way in which these mechanisms, when acting simultaneously, contribute to heat dissipation is intriguing, and needs to be further explored in subterranean rodents and mammals in general.

Considering the effect of sociality on heat dissipation, Šumbera et al.^31^ found that social *F. mechowii* had higher T_s_ on both the venter and the dorsum, but not the feet compared with solitary *H. argenteocinereus*. Our results partially support the effect of sociality on heat dissipation in larger number of species. As expected, the role of the feet in heat dissipation was independent of social organization, as demonstrated by almost identical regression lines for solitary and social species (Fig. 4C). On the contrary, solitary species had significantly higher T_diff_ on the dorsum across tested T_a_s, especially at lower temperatures (Fig. 4A). Solitary species thus conserve heat more effectively through this body region especially at lower T_a_s (cf.^31^). Although the mean ventral T_diff_ of solitary species was about 2 °C higher across experimental T_a_s (Fig. 4B), this difference only approached significance. This might be caused mainly by a large intraspecific variability of the ventral T_diff_ among the four studied social species (compare Fig. 4A,B). Indeed, *S. cyanus* and *C. hottentotus* had markedly higher T_diff_ than two *Fukomys* species at the lowest T_a_ of 10 °C (see Fig. 3). We may speculate that such difference can indicate better ventral fur insulation in these two species, as they occupy harsher environmental conditions compared to tropical habitats of both *Fukomys* species (see Table 1). Further studies that will focus on the fur quality including more species from various environmental conditions could help to better understand this concept.

Higher heat dissipation from the surface of social subterranean species is linked with lower insulative properties of their fur^31,33^. Dense heat conserving fur is probably not necessary in social species because they can take advantage of social thermoregulation at low T_a_s^37,38^. While individuals of social species are temporarily single such as during digging, burrow maintaining, and patrolling, metabolic heat produced by their activities could help to keep a stable T_b_ (c.f.^36,78^). On the contrary, solitary species cannot use huddling. Instead, solitary species can minimise body surfaces exposed to the environment solely by curling up during the rest, as observed in all solitary species at low T_a_s in the present study, and also during several radio-tracking studies under natural conditions^79–81^. Denser and thus well insulating fur across the dorsum and flanks of solitary species^31,33^ is important because this body region is always exposed to the environment when the animal is rolled into a ball. On the other hand, less insulating fur in social species is advantageous at high T_a_s, or whilst digging when it is necessary to dissipate surplus metabolic heat to avoid overheating. In this situation, better heat conserving fur of solitary species complicates heat dissipation as we observed higher T_diff_ on the dorsum, and in some species also on the venter, compared to the feet at the highest studied T_a_ in solitary species only (see Supplementary Table S2). Thus, their feet likely play a crucial role in heat dissipation at high T_a_ and/or during digging in solitary species.

Different social organisation and fur characteristics resulting in different body heat dissipation may have relevant ecological consequences. For the naked mole-rat *Heterocephalus glaber*, the combination of small body mass and hairless surface resulting together in very high heat losses limits its occurrence in lowland areas of East Africa because it is not able to disperse through the cooler highlands in the western and northern borders of its distribution^82^. We may assume that higher heat losses in social species may handicap them during dispersal and forming of new families especially in colder climate if they disperse singly. Although it is very difficult to collect such information in nature, it seems that dispersing individuals of social species can stay alone for long period of time, likely for weeks, months, or even years^45–48^ facing high thermal challenges during that time. In this regard, although anecdotal, the radio-tracking of *F. mechowii* revealed interesting finding related to this phenomenon^47^. Lövy et al.^47^ found that a solitarily living female had a different circadian activity pattern and resting position than conspecific mole-rats living in a group. This female was most active during the hottest part of day, and usually rested in the curled-up body position while mole-rats from the family were most active during the night, and rested communally outstretched in their nest as indicated by a signal of their collars. Both changing of resting position and activity pattern likely minimised her heat losses. Interestingly, the peak occurred during the hottest part of the day and the pattern of its activity was more similar to that of the activity of the solitary *H. argenteocinereus* and *N. galili*^79,80^ than to *F. mechowii* living in a family group^47^. Besides, less insulating fur of social species could explain why the social *F. whytei* does not colonise generally colder Afromontane grasslands from the lower altitudes of the Nyika Plateau where it is common in contrast to the solitary *H. argenteocinereus*, even though there is a higher food supply and more easily workable soil in grasslands^44^.

Finally, we analysed whether T_diff_ differs along the venter, and if sociality influences the heterogeneity of ventral T_diff_ distribution as suggested for two mole-rat species with contrasting sociality^31^. For this purpose, we divided the venter into five areas (Fig. 1), and for each species, we analysed the pattern of T_diff_ at T_a_ below, within, and above TNZ (Table 5). Although there were some statistically significant differences in the pattern of T_diff_ among these ventral areas for particular species at T_a_s below and within TNZ (except for *B. suillus* and *G. capensis*; see Table 5 and Supplementary Fig. S1), the differences were usually small. Due to this and the absence of any consistent pattern within the tested species, we think that the differences have probably low biological importance. In *C. hottentotus* and *S. cyanus*, the differences were found only within TNZ, but not in the lowest T_a_. As expected, we found homogeneous pattern of T_diff_ at the highest T_a_. Together with low values of T_diff_ at this T_a_, it demonstrates that heat dissipation through the ventral surface is maximal. Similarly, no consistent differences in the pattern of the T_diff_ change across the five ventral areas between solitary and social species were detected in either of the three T_a_s (Table 5).

Nevertheless, limitations of the IRT approach should be considered when measuring T_s_ of restricted body areas in small mammals, such as those five ventral areas explored in our study. For instance, the fact that the animals move and bend their bodies during the short recording might provide relatively variable data for such small areas. In this case, the analysis of the insulative quality of the fur might be a better approach to reveal potential differences if such heterogeneity exists. It should be noted that recent findings based on the histological analysis of small skin patches along the body in *F. mechowii* did not reveal any differences in the amount and structure of the adipose tissue and vascularisation along its venter^43^. This suggests fur having the main role in heat dissipation through the integument in mole-rats and subterranean rodents generally.

For future research on heat dissipation in subterranean rodents, histological analysis of the feet, analysis of T_s_ of the only hairless rodent *H. glaber*, experiments based on fur shaving, direct measurement of thermal insulation of fur, and/or measuring of skin temperature with thermocouples could bring valuable information on how subterranean rodents handle heat dissipation which is a crucial aspect for mammals who frequent burrows for their whole lives.

## Supplementary Information

Supplementary Information.

## Data Availability

The datasets generated and analysed during the current study are available from the corresponding author on reasonable request.
