# Supplementary material for: Heat dissipation in subterranean rodents: the role of body region and social organisation
Source: Sci Rep. 2021 Jan 21;11:2029. doi: 10.1038/s41598-021-81404-3 (PMC7820455; doi:10.1038/s41598-021-81404-3)
Supplement: Supplementary file 1 — Supplementary Information. [file 41598_2021_81404_MOESM1_ESM.docx]

**Heat dissipation in subterranean rodents: The role of body region and social organisation**

František Vejmělka, Jan Okrouhlík, Matěj Lövy, Gabriel Šaffa, Eviatar Nevo, Nigel Charles Bennett, Radim Šumbera^*^

email address of the corresponding author^*^: sumbera@prf.jcu.cz

**
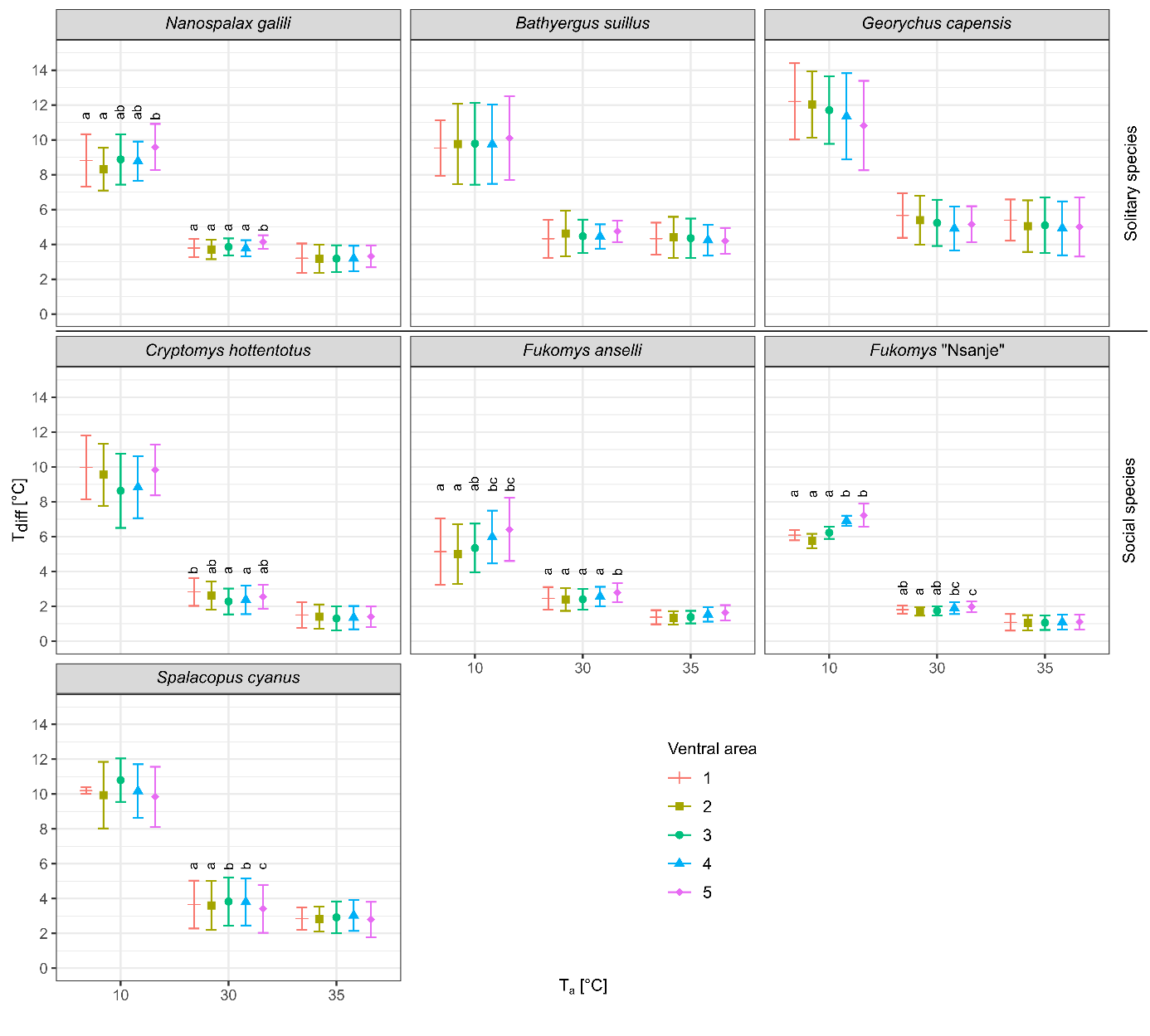
Figure S1.** The difference between core body temperature and surface temperature (T_diff_) for five areas of the venter at the T_a_ of 10, 30 and 35°C in seven species of subterranean rodents (means ± SD are depicted). Differences among five ventral areas for each species were computed by post-hoc Tukey’s tests; any two ventral areas are significantly different at p < 0.05 in case they do not have any letters in common. The figure was prepared using the program Inkscape 0.92 (https://inkscape.org/).

**
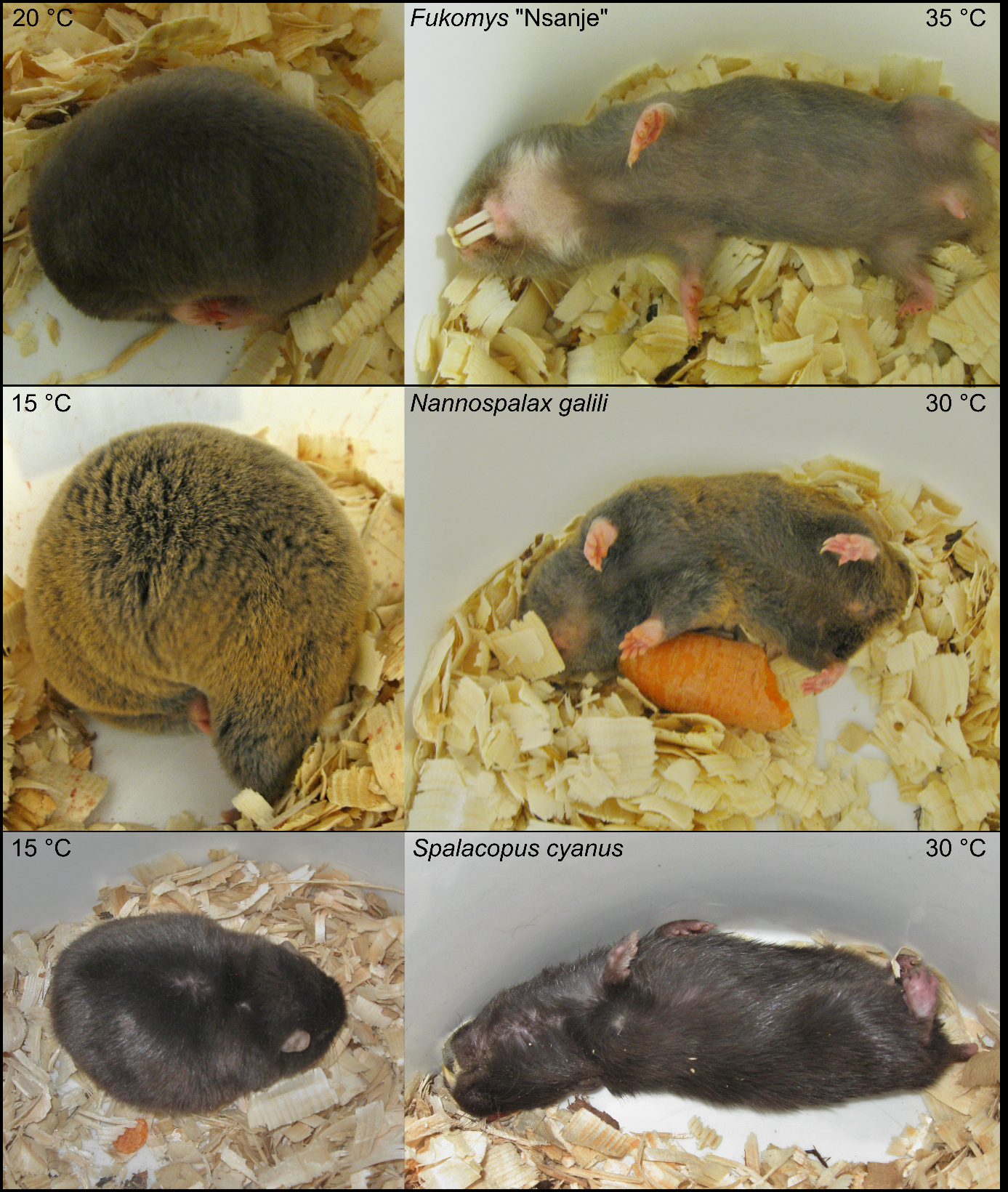
**

**Figure S2.** Examples of body postures in three species under the study observed during the acclimation period prior to the measuring of T_b_ and T_s_ at low (left photos) and high (right photos) ambient temperatures (T_a_s). The figure was prepared using the program Inkscape 0.92 (https://inkscape.org/).

**Table S1.** Core body temperature (T_b_) and surface temperatures (T_s_) of the venter, dorsum and feet measured across the range of ambient temperatures (T_a_s) in seven subterranean rodent species (n denotes to the number of individuals tested for each species in each T_a_; means ± SD are depicted).

| **Species** | **Temperature** | **T_a_ [°C]** | | | | | |
| --- | --- | --- | --- | --- | --- | --- | --- |
|  |  | **10 (n)** | **15 (n)** | **20 (n)** | **25 (n)** | **30 (n)** | **35 (n)** |
| *B. suillus* | Core body | 34.4±1.0 (10) | 34.5±0.4 (10) | 34.7±0.6 (10) | 35.1±0.6 (10) | 35.9±0.6 (10) | 37.4±0.6 (10) |
| *G. capensis* | Core body | 35.1±0.9 (9) | 35.2±0.8 (9) | 35.3±1.0 (9) | 36.0±0.6 (9) | 36.3±0.5 (9) | 38.0±0.8 (9) |
| *C. hottentotus* | Core body | 33.8±1.4 (10) | 33.9±0.6 (10) | 34.4±0.6 (10) | 34.6±0.5 (10) | 35.2±0.6 (10) | 37.6±0.7 (10) |
| *F. anselli* | Core body | 33.8±1.0 (9) | 34.6±0.2 (9) | 34.9±0.5 (9) | 35.1±0.5 (9) | 35.2±0.3 (9) | 37.6±0.3 (9) |
| *F.* “Nsanje” | Core body | 34.0±0.3 (10) | 34.1±0.4 (10) | 33.7±0.7 (10) | 33.6±0.3 (10) | 35.2±0.2 (10) | 37.7±0.4 (10) |
| *S. cyanus* | Core body | 35.4±0.2 (5) | 35.4±0.5 (5) | 35.6±0.7 (5) | 35.8±0.4 (5) | 37.7±0.3 (5) | 38.9±0.2 (5) |
| *N. galili* | Core body | 35.6±0.6 (20) | 35.7±0.6 (20) | 36.3±0.7 (20) | 36.4±0.7 (20) | 37.3±0.4 (20) | 38.5±0.4 (20) |
| *B. suillus* | Dorsum | 17.6±1.0 (5) | 21.2±1.0 (5) | 23.6±0.8 (5) | 27.9±1.2 (3) | 31.7±0.5 (5) | 34.0±1.0 (7) |
| *G. capensis* | Dorsum | 16.1±0.8 (4) | 20.3±0.7 (5) | 24.1±0.9 (3) | 26.3±0.5 (3) | 29.1±0.9 (4) | 33.5±1.4 (5) |
| *C. hottentotus* | Dorsum | 20.3±0.6 (3) | 24.2±0.6 (3) | 26.5±0.3 (4) | 29.2±0.5 (4) | 32.4±0.4 (3) | 36.4±0.4 (4) |
| *F. anselli* | Dorsum | 24.9±1.0 (4) | 27.1±0.7 (6) | 28.8±0.4 (4) | 30.5±0.6 (6) | 32.3±0.3 (6) | 36.3±0.4 (4) |
| *F.* “Nsanje” | Dorsum | 22.6±0.5 (2) | 24.9±0.7 (4) | 27.5±0.7 (3) | 29.2±0.3 (5) | 32.6±0.3 (5) | 36.2±0.5 (7) |
| *S. cyanus* | Dorsum | 21.2±3.7 (3) | 24.3±0.4 (4) | 27.7±1.0 (2) | 30.1±0.6 (3) | 33.3±1.7 (2) | 35.5±1.4 (3) |
| *N. galili* | Dorsum | 20.2±1.4 (10) | 23.3±1.1 (13) | 26.7±0.6 (8) | 28.8±0.8 (10) | 32.3±0.8 (10) | 34.5±1.1 (8) |
| *B. suillus* | Venter | 24.6±1.7 (5) | 26.2±1.2 (5) | 27.6±0.9 (5) | 30.0±1.2 (3) | 31.4±0.7 (5) | 33.1±1.2 (7) |
| *G. capensis* | Venter | 23.0±2.2 (4) | 25.7±1.3 (5) | 28.2±1.0 (3) | 29.9±0.8 (3) | 30.6±1.4 (4) | 33.6±1.0 (5) |
| *C. hottentotus* | Venter | 24.5±1.1 (3) | 27.0±0.4 (3) | 28.8±0.5 (4) | 30.5±0.4 (4) | 32.6±0.8 (3) | 36.2±0.7 (4) |
| *F. anselli* | Venter | 28.4±1.4 (4) | 29.7±1.5 (6) | 30.6±0.8 (4) | 32.0±0.8 (6) | 33.0±0.6 (6) | 36.1±0.4 (4) |
| *F.* “Nsanje” | Venter | 27.4±0.4 (2) | 28.7±0.6 (4) | 30.0±0.9 (3) | 30.7±0.4 (5) | 33.3±0.3 (5) | 36.8±0.4 (7) |
| *S. cyanus* | Venter | 24.4±3.5 (3) | 25.9±0.6 (4) | 29.2±1.6 (2) | 31.1±0.9 (3) | 33.5±1.7 (2) | 36.0±1.3 (3) |
| *N. galili* | Venter | 26.8±1.2 (10) | 28.3±0.9 (13) | 30.2±0.7 (8) | 31.4±0.7 (10) | 33.4±0.6 (10) | 35.2±0.9 (8) |
| *B. suillus* | Feet | 18.9±6.2 (5) | 24.6±4.0 (5) | 28.7±3.2 (5) | 30.7±2.6 (3) | 34.4±0.4 (5) | 36.4±0.6 (7) |
| *G. capensis* | Feet | 19.8±4.1 (4) | 22.7±2.4 (5) | 25.7±2.0 (3) | 27.5±0.6 (3) | 31.9±0.5 (4) | 36.3±0.7 (5) |
| *C. hottentotus* | Feet | 20.8±5.8 (3) | 21.5±4.8 (3) | 24.1±3.5 (4) | 26.8±1.4 (4) | 31.0±1.9 (3) | 36.9±0.6 (4) |
| *F. anselli* | Feet | 18.6±1.2 (4) | 21.1±1.4 (6) | 23.3±0.2 (4) | 27.1±0.8 (6) | 31.3±0.9 (6) | 36.3±0.7 (4) |
| *F.* “Nsanje” | Feet | 17.7±0.3 (2) | 21.3±4.0 (4) | 24.2±1.1 (3) | 28.1±0.5 (5) | 32.4±0.7 (5) | 36.3±0.7 (7) |
| *S. cyanus* | Feet | 17.0±1.7 (3) | 20.0±1.1 (4) | 25.9±1.0 (2) | 28.3±0.6 (3) | 33.5±0.9 (2) | 36.1±0.5 (3) |
| *N. galili* | Feet | 15.5±2.9 (10) | 21.2±1.7 (13) | 24.8±1.9 (8) | 28.6±1.4 (10) | 33.4±0.4 (10) | 35.8±0.8 (8) |

**Table S2.** The results of GLS models comparing T_diff_ values among dorsum, venter, and feet at the T_a_ of 10, 30, and 35 °C in each of the seven subterranean rodent species (D – dorsum, V – venter, F – feet; statistically significant results among the three body regions after the Bonferroni procedure p < 0.0024 are marked with an asterisk; D × F, V × D, and V × F show *post-hoc* comparisons between the pairs of body regions significant after the Bonferroni procedure p < 0.0038, for details see Methods).

| **Species** | **T_a_** | **Body region (D-V-F)** | **D** × **F** | **V** × **D** | **V** × **F** |
| --- | --- | --- | --- | --- | --- |
| *B. suillus* | 10 | <0.0001 (60.4)* |  | <0.001 |  |
|  | 30 | <0.0001 (50.6)* | <0.0001 |  | <0.0001 |
|  | 35 | <0.0001 (80.7)* | <0.0001 |  | <0.0001 |
| *G. capensis* | 10 | <0.0001 (48.5)* |  | <0.001 |  |
|  | 30 | 0.0036 (11.2) |  |  |  |
|  | 35 | <0.0001 (14.3)* | <0.0001 |  | <0.0001 |
| *C. hottentotus* | 10 | <0.0001 (59.2)* |  | <0.001 |  |
|  | 30 | 0.504 (0.8) |  |  |  |
|  | 35 | 0.0095 (8.2) |  |  |  |
| *F. anselli* | 10 | <0.0001 (116.6)* | <0.0001 | <0.0001 | <0.0001 |
|  | 30 | <0.0001 (61.7)* | <0.0001 | 0.0006 | <0.0001 |
|  | 35 | 0.06 (3.9) |  |  |  |
| *F.* “Nsanje” | 10 | <0.0001 (294.2)* | <0.0001 | <0.0001 | <0.0001 |
|  | 30 | 0.009 (7.1) |  |  |  |
|  | 35 | 0.0044 (7.5) |  |  |  |
| *S. cyanus* | 10 | <0.0001 (85.7)* | <0.0001 | <0.0001 | <0.0001 |
|  | 30 | 0.062 (8.0) |  |  |  |
|  | 35 | 0.019 (8.2) |  |  |  |
| *N. galili* | 10 | <0.0001(205.8)* | <0.0001 | <0.0001 | <0.0001 |
|  | 30 | <0.0001 (31.8)* | <0.0001 | <0.0001 |  |
|  | 35 | <0.0001 (45.1)* | <0.0001 | <0.0001 | <0.0001 |
